# Ovarian lymphoma

**DOI:** 10.4103/0971-5851.56333

**Published:** 2009

**Authors:** Julian A. Crasta, Elizabeth Vallikad

**Affiliations:** *Department of Pathology, St. John′s Medical College, Bangalore, Karnataka, India*; *Department of Gynecologic Oncology, St. John′s Medical College, Bangalore, Karnataka, India*

**Keywords:** *Ovary*, *lymphoma*

## Abstract

The involvement of the ovary in lymphomatous processes is rare. Such an involvement can occur in 2 ways, either primary or secondary, which usually presents with abdominal or pelvic complaints. We present a case of secondary involvement of the ovary with occult extra-ovarian nodal disease and discuss the histogenesis of ovarian lymphomas with criteria for diagnosis and differential diagnosis.

## INTRODUCTION

The involvement of the ovary in lymphomatous processes is rare, but ovary is the common site in the female genital tract to be involved by the hematological malignancies.[1] The occurrence of lymphomas primarily arising in the ovaries has long been debated since no lymphoid tissue is found in the ovaries.[2] Involvement of the ovary by malignant lymphoma can be primary or secondary and is discovered incidentally during a workup for abdominal or pelvic complaints.[3,4]We present a case of ovarian lymphoma presenting as an ovarian mass with gastrointestinal symptoms with occult extra-ovarian disease in this article.

## CASE REPORT

A 44-year-old woman presented with complaints of increasing pain in the abdomen and dyspepsia of 3 months′ duration. Physical examination revealed a tender mass in the right iliac fossa, which appeared to be arising from the pelvis and extending up to the umbilicus. Per vaginal examination revealed a mass in the pouch of Douglas pushing the uterus to the left side.

An abdomino-pelvic ultrasound revealed a right ovarian mass with a bowel mass in the right iliac fossa. The patient underwent a colonoscopy for evaluation of the bowel mass, which revealed a large submucosal nodule with ulceration occupying half the circumference and constricting the lumen. Biopsy revealed an inflammatory infiltrate in the lamina propria with much crush artifact, and no definite diagnosis was possible. All the tumor markers were within normal limits. A provisional diagnosis of right ovarian tumor with mass in right iliac fossa was made. Exploratory laparotomy revealed a well-encapsulated, intact right ovarian tumor. The mass in the right iliac fossa appeared to be an inflammatory mass with pus in the pericolic gutter, thickening of the bowel wall and adherent omentum. Perioperative diagnosis of right ovarian mass with right pericolic abscess was made. Abscess drainage with right salpingo-oophorectomy and omenectomy was performed.

**Figure 1:**
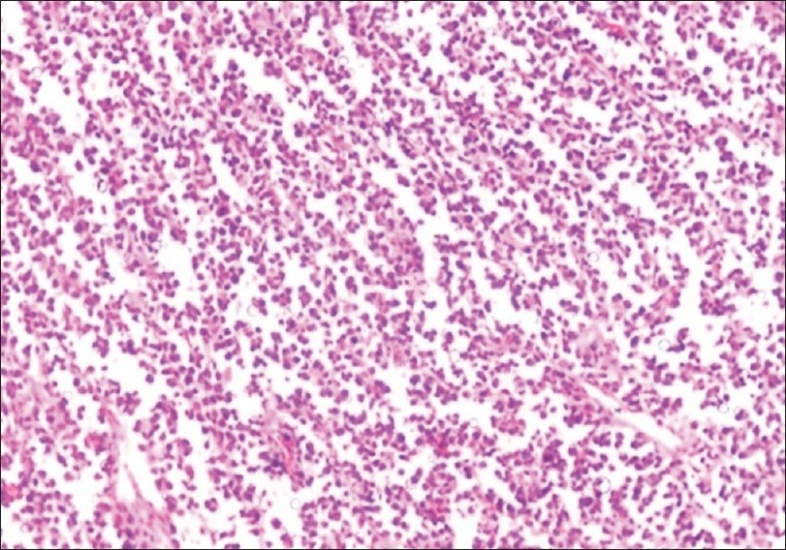
Diffuse sheets of monotonous medium-sized cells (×10)

**Figure 2:**
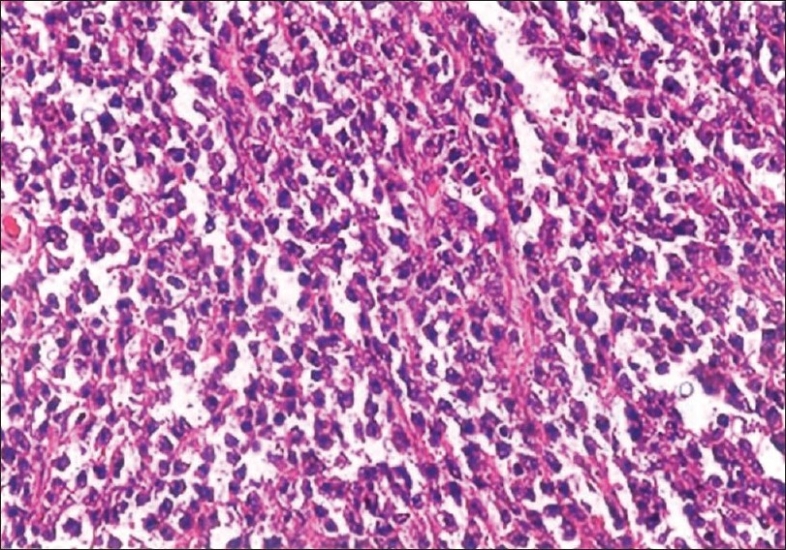
Cells with hyperchromatic nuclei and scant cytoplasm with dispersed large cells (×45)

On gross examination, the right ovarian mass measured 8.5 × 6.0 × 3.0 cm with an intact capsule and was solid and grayish white. The omentum and the fibro-adipose tissue from the paracolic gutter were covered by exudates and showed no nodules. Microscopic examination of ovarian mass revealed ovarian tissue was replaced by diffuse sheets of monotonous medium-sized cells, with a few large cells [Figure 1] at foci forming cords and trabeculae. The cells had scant cytoplasm and cleaved nuclei-clumped chromatin with conspicuous nucleoli [Figure 2]. There was increased mitotic activity with many atypical mitoses. Neither follicular structures nor starry-sky pattern was noted. Immunohistochemically, the cells were positive for LCA and negative for cytokeratin, which confirmed the diagnosis of lymphoma. Sections from the omentum and biopsy from the paracolic gutter revealed focal collections of these lymphoid cells with areas of necrosis and acute inflammatory infiltrate. A diagnosis of diffuse non-Hodgkin′s lymphoma (probably diffuse large-cell lymphoma) involving the ovary, omentum and pericolic tissue was rendered.

The patient was evaluated postoperatively with an abdominal CT scan, which revealed multiple enlarged lymph nodes involving the common iliac, internal iliac and external iliac groups. Bone marrow aspirates and trephine biopsy ruled out any marrow involvement. A diagnosis of stage IV (Ann Arbor staging) non-Hodgkin′s lymphoma with secondary ovarian involvement with an initial clinical presentation of occult extra-ovarian disease was made. A chemotherapy protocol comprising of 6 cycles of CHOP regime was administered to the patient, but the patient did not respond well to the therapy and had a progressive disease. She presented with para-aortic and splenic hilar lymphadenopathy with involvement of left kidney at the end of the sixth cycle. External radiation of 3000 cGy was given to the para-aortic area. The patient was admitted to another oncology center and was lost to further follow-up.

## DISCUSSION

Malignant lymphoid tumors of the female genital tract are unusual, although the ovaries are the most common sites to be affected, with up to 25% of women dying with lymphomas having ovarian involvement.[1] The ovarian involvement in malignant lymphoma may be primary or secondary. The secondary involvement may be of 2 types: (1) as an initial clinical presentation of occult extra-ovarian disease or (2) as a manifestation of widely disseminated disease.[1] The distinction is of considerable importance because primary extra-nodal lymphomas run a less aggressive course and have a 5-year survival rate of 80% as compared to the malignant lymphomas, which have a 5-year survival rate of only 33%.[4]

Lymphomas of the ovary may occur at any age, but mostly women in their 40s are affected.[3-5] Lymphomas of the ovary, whether primary or secondary, may have varied presentation, most of them being discovered incidentally during the workup for pelvic and abdominal complaints with an abdominal or pelvic mass.[3-5] The patient in the present case presented with gastrointestinal symptoms and was found to have a mass in the POD.

It is at times difficult to ascertain whether lymphomas of the ovary are primary or secondary. The definition of primary ovarian lymphoma has been a subject of controversy for years. Skodras *et al*.,[6] based on the data derived from 15 cases of primary ovarian lymphoma, have proposed that the designation of an ovarian lymphoma as primary should require the following: (1) There is presence of an ovarian mass, confined to one or both ovaries; the lymphoma should be considered as an ovarian primary even if only microscopic involvement of the contiguous lymph nodes is detected. Talerman [7]adds that if a local spread from ovary to adjacent structures is present, this should not preclude the diagnosis. (2) Extensive intraoperative and postoperative staging procedures show no evidence of lymphoma elsewhere in the body. However, Paladugu *et al*.,[8] thought the criteria were insufficiently stringent and proposed that there should be a disease-free interval of at least 60 months after the oophorectomy.

As the clinical presentation of our patient was with no evidence of generalized lymphadenopathy preoperatively, a diagnosis of primary ovarian lymphoma was considered. However, on subsequent CT evaluation and multiple abdominal lymph node involvement, a diagnosis of secondary ovarian lymphoma with an initial ovarian presentation of occult extra-ovarian disease was considered, and the patient was treated accordingly.

Ferrozzi *et al*.,[9] describe the typical features of ovarian lymphoma on imaging. CT scan will show hypodense lesions with mild contrast enhancement, whereas ultrasound will be nonspecific with hypoechoic patterns. MRI will show them as homogenous masses that have hypointense T1-weighted images and slightly hyperintense T2-weighted images. The authors opine that a diagnosis of ovarian lymphoma may be considered when the bilateral ovarian tumor appears homogenous in the absence of ascites.

The histological appearances of lymphoma in the ovary are generally similar to those seen in the extra-ovarian sites. In the ovary, however, there is a great tendency for the tumor cells to grow in cords and nests, appearing to cling to the reticulin, forming pseudoacini.[10] The most common types of lymphomas encountered in the ovary are diffuse large-cell, Burkitt and follicular lymphomas.[10] Rarely precursor B-cell lymphoblastic lymphomas are also encountered.[11,12] However, these need to be distinguished from other round-cell tumors such as metastatic poorly differentiated carcinoma, especially of mammary origin; primary small-cell carcinoma; adult granulosa cell tumor; and dysgerminoma. Immunohistochemical studies will help to distinguish between these tumors.[10]

It is obvious that origin of lymphomas in the ovary requires the presence of preexisting benign lymphoid infiltrate in the ovary. Nelson *et al*.,[2] doubt the validity of a primary lesion since ovarian lymphocytic aggregates are not present. Skodras *et al*.,[6] studied 37 oophorectomy specimens and confirmed immunohistochemically that ovaries contain scattered lymphocytes or small lymphoid aggregates. They suggest that the reactive lymphocytes can secondarily populate the ovary in response to various ovarian lesions, including PID, endometriosis, benign/malignant neoplasm, the very common leutin, follicular and surface inclusion cysts. It seems, on rare occasions these lymphocytic populations may undergo malignant change and give rise to POL. However, there are many cases in which there is no clinical or histological evidence of inflammation.[4]
